# Evidence for Immune Response, Axonal Dysfunction and Reduced Endocytosis in the Substantia Nigra in Early Stage Parkinson’s Disease

**DOI:** 10.1371/journal.pone.0128651

**Published:** 2015-06-18

**Authors:** Anke A. Dijkstra, Angela Ingrassia, Renee X. de Menezes, Ronald E. van Kesteren, Annemieke J. M. Rozemuller, Peter Heutink, Wilma D. J. van de Berg

**Affiliations:** 1 Department of Anatomy and Neurosciences, section Quantitative Morphology, VU University Medical Center, Neuroscience Campus Amsterdam, Amsterdam, The Netherlands; 2 Department of Epidemiology and Biostatistics, VU University Medical Center, Neuroscience Campus Amsterdam, Amsterdam, The Netherlands; 3 Center for Neurogenomics and Cognitive Research, VU University Medical Center, Neuroscience Campus Amsterdam, Amsterdam, The Netherlands; 4 Department of Pathology, VU University Medical Center, Neuroscience Campus Amsterdam, Amsterdam, The Netherlands; 5 Department of Medical genomics, VU University Medical Center, Neuroscience Campus Amsterdam, Amsterdam, The Netherlands; 6 German Center for Neurodegenerative diseases (DZNE), Tübingen, Germany; UCL Institute of Neurology, UNITED KINGDOM

## Abstract

Subjects with incidental Lewy body disease (iLBD) may represent the premotor stage of Parkinson’s disease (PD). To elucidate molecular mechanisms underlying neuronal dysfunction and alpha-synuclein pathology in the premotor phase of PD, we investigated the transcriptome of the substantia nigra (SN) of well-characterized iLBD, PD donors and age-matched controls with Braak alpha-synuclein stage ranging from 0–6. In Braak alpha-synuclein stages 1 and 2, we observed deregulation of pathways linked to axonal degeneration, immune response and endocytosis, including axonal guidance signaling, mTOR signaling, EIF2 signaling and clathrin-mediated endocytosis in the SN. In Braak stages 3 and 4, we observed deregulation of pathways involved in protein translation and cell survival, including mTOR and EIF2 signaling. In Braak stages 5 and 6, we observed deregulation of dopaminergic signaling, axonal guidance signaling and thrombin signaling. Throughout the progression of PD pathology, we observed a deregulation of mTOR, EIF2 and regulation of eIF4 and p70S6K signaling in the SN. Our results indicate that molecular mechanisms related to axonal dysfunction, endocytosis and immune response are an early event in PD pathology, whereas mTOR and EIF2 signaling are impaired throughout disease progression. These pathways may hold the key to altering the disease progression in PD.

## Introduction

Substantial dopaminergic cell loss in the substantia nigra (SN) is considered to be the pathoanatomical substrate of the motor symptoms in Parkinson’s disease (PD) [1]. The neuronal loss in the SN is accompanied by the presence of Lewy bodies (LBs) and Lewy neurites (LNs), which are abnormal protein aggregates, mainly consisting of misfolded alpha-synuclein [2]. The alpha-synuclein pathology in PD is however not limited to the SN, but observed in many brain regions [3–6]. It has been postulated that the alpha-synuclein pathology in the brain starts in the lower brainstem and then advances to the limbic and neocortical brain regions during progression of the disease [6–8]. Alpha-synuclein pathology and nigrostriatal loss have been observed in aged individuals without evidence of Parkinsonism or dementia during life [9, 10] and are defined pathologically as incidental Lewy Body (iLBD) subjects, suggesting that these subjects may represent the premotor stage of PD [11–14]. Studying post-mortem SN tissue of iLBD subjects may, therefore, provide insight into molecular mechanisms involved in alpha-synuclein aggregation and neuronal dysfunction in early stage PD and shed light on its pathogenesis.

The etiology of PD remains largely unknown, but the role of genetic factors in PD development has been firmly established during the past decade with the identification of 16 ‘PARK’ loci [15]. These loci have mainly been identified in patients with familial PD, but also patients with sporadic PD, using Genome Wide Association Studies (GWAS) [16, 17]. The identified genes have provided useful insight in the molecular pathways that contribute to the pathogenesis of PD [18]. In addition, advanced genomics and proteomics techniques have been applied to discover the molecular signature of sporadic PD. For example, transcriptome analysis of brain structures, such as striatum, SN and locus coeruleus (LC) of PD patients and controls revealed changes in the expression of genes involved in a variety of pathways and cellular processes, including ubiquitination and proteasomal degradation of proteins, oxidative stress, vesicle trafficking, cytoskeletal stability, axonal guidance, dopamine neurotransmission and metabolism, neurotrophic signaling, inflammation and programmed cell death [19–33]. The involvement of the mitochondrial dysfunction and synaptic loss in the pathogenesis of PD has been successfully confirmed in animal and *in vitro* models in PD (for review: [34, 35]). However, it is still unknown if these processes contribute to the early stages of PD, in particular the premotor stage.

In the present study, we aimed to identify molecular pathways that play a key role in the progression of alpha-synuclein pathology by studying the transcriptome of the post-mortem SN of well-characterized PD patients, iLBD subjects and age-matched controls using microarray analysis. To elucidate the mechanisms underlying the alpha-synuclein aggregation and nigral neuronal death in early stage PD, we focused on transcriptome changes prior to appearance of alpha-synuclein aggregates in the SN, in Braak alpha-synuclein stage 1 and 2, and those affected during disease progression in PD.

## Materials and Methods

### Post-mortem human SN tissue

Snap-frozen post-mortem SN tissue was obtained from the Netherlands Brainbank (NBB) and the department of Pathology of VU University Medical Center (VUmc, Amsterdam, The Netherlands). All donors had given informed consent for using the brain tissue and the extensive neuropathological and clinical information for scientific research, in compliance with ethical and legal guidelines. Records including a summary of medical history were available of all patients and controls included in this study.

All subjects were neuropathologically evaluated by experts (AJMR, WK and WvB) and classified for Braak Alzheimer (neurofibrillary tangles; NFTs score 0-VI [36]; amyloid-beta plaques stage 0-C [37]) and Braak alpha-synuclein stages 0–6 [3]. The distribution and density of NFTs were determined using Bodian staining and immunohistochemistry for hyperphosphorylated tau (Clone AT-8; dilution 1:300 pre-treatment in Tris-buffered saline pH 9.0 in the microwave, Innogenetics, Belgium), and the alpha-synuclein pathology was determined routinely using immunohistochemistry for alpha-synuclein in several sections throughout the assessed structures by two investigators (WK, WvB) (KM51 antibody, Novocastra, Newcastle upon Tyne, UK; dilution 1:500; pre-treatment: citrate buffer (pH 6.0) in the microwave and 80% formic acid). Senile plaques were immunohistochemically stained for amyloid-beta (clone M0872, dilution 1:500; DAKO, Denmark pre-treatment: citrate buffer (pH 6.0) in the microwave and 80% formic acid).

We used stringent inclusion and exclusion criteria, based on clinical diagnosis and comprehensive pathological assessment, for selecting controls, iLBD subjects and PD donors. In total, 28 donors with Braak alpha-synuclein stages ranging from 0 to 6 were included in the present study; 11 iLBD subjects, 9 clinically diagnosed and pathologically confirmed PD donors and 8 age-matched non-demented controls. Age-matched non-demented controls had no history of neurological or psychiatric disorders. ILBD subjects had no history of neurological or psychiatric disorders and revealed alpha-synuclein pathology in the medulla oblongata (MO) and olfactory bulb (OB) (Braak alpha-synuclein stage 1) or MO, OB and LC (Braak alpha-synuclein stage 2) or MO, LC and SN (Braak alpha-synuclein stage 3). PD patients were clinically diagnosed with PD or PD with dementia (PDD) by neurologists and/or geriatricians and had no history of cancer or concomitant disease of the central nervous system. The distribution pattern of the PD patients was consistent with Braak alpha-synuclein stage 4–6. ILBD subjects and PD patients in which the distribution pattern of alpha-synuclein pathology was atypical at autopsy were excluded as well as donors with concomitant pathologies [5, 38, 39]. Only sporadic PD patients with an age of onset > 45 years of age were included. The demographic data, including mean age at death, gender, and Braak stages for PD and Alzheimer’s disease pathology of the PD patients, iLBD subjects and controls are listed in Table 1. A detailed overview of all the demographic, clinical and pathological data including of all subjects included in the present study is given in S1 Table.

**Table 1:** Clinical and pathological characteristics and mean neuromelanin-containing cell density of donors included in the study.

	Control: Braak alpha-synuclein stage 0	Braak alpha-synuclein stage 1–2	Braak alpha-synuclein stage 3–4	PD Braak alpha-synuclein stage 5–6	
	(n = 8)	(n = 5)	(n = 7)	(n = 8)	p-value
Gender					
Male, n (%)	4 (50%)	2 (40%)	4 (57%)	4 (50%)	0.95^b^
Female, n (%)	4 (50%)	3 (60%)	3 (43%)	4 (50%)	
Age of onset (SD)	NA	NA	50 (n = 1)	63.6 (11.2)	
PD duration (SD)	NA	NA	24 (n = 1)	17.7 (6.7)	
Estimated H&Y, median (range)	NA	NA	4.0 (n = 1)	5.0 (4.0–5.0)	
Demented, n (%)	NA	NA	100% (n = 1)	4 (50%)	
Age at death, y (SD)	75.5 (7.6)	79.4 (7.1)	78.8(8.5)	78.5 (8.5)	0.81^a^
Tangle score, median (range)	1(0–2)	2(0–2)	1 (0–2)	1 (0–2)	0.76^b^
Amyloid-beta score, median (range)	0 (0-B)	A (0-B)	0 (0-C)	A (0-C)	0.38^b^
Neuronal cell density, mean (standard deviation)	6659 (960)	5022 (930)	4682 (1440)	2575 (530)	<0.001^a^

Abbreviations: iLBD: incidental Lewy body disease; PD = Parkinson’s disease; H&Y = Hoehn & Yahr.

^a^ANOVA;

^b^Kruskal-Wallis. NA = not available

### Tissue processing for microarray analysis and morphometry

The SN was identified macroscopically in snap-frozen post-mortem tissue of the mesencephalon and subsequently dissected in a cryostat (-18°C). The SN was marked and serial 40-μm-thick sections were made to microscopically verify that all neuromelanin-containing neurons of the SN were located within the punch. Every 10th section collected throughout the SN of all donors was used to estimate the number of neuromelanin-containing neurons in the SN using the optical fractionator method [40], as described in previously [41]. The mean neuromelanin-containing cell density is listed in Table 1. The average neuromelanin-containing neuron density in the SN of the controls was 6659 (sd = 960), Braak alpha-synuclein stage 1 and 2 was 5022 (sd = 930); Braak alpha-synuclein stage 3 and 4: 4682 (sd = 1440) and Braak alpha-synuclein stage 5–6: 2575 (sd = 530). An ANOVA revealed that the cell density between groups is statistically different (F(3) = 19.57, p<0.001). Punches were collected throughout the entire SN in 2mL RNAse free Eppendorf tubes. Total RNA was isolated with a Trizol Reagent (Invitrogen, Carlsbad, CA, USA)/chloroform protocol. Frozen SN punches were homogenized in Trizol reagent with an IKA Ultra-Turrax T 18 basic homogenizer. Chloroform was added to the tubes, and after vortexing, samples were left at room temperature for 3 minutes, then spinned at 13000 rpm for 15 minutes at 4°C. After transferring the supernatants to new tubes and adding linear acryl amide and iso-propanol, samples were kept at room temperature for 10 minutes and then spinned for 10 minutes at 13.000 rpm at 4°C. Pellets were washed and spinned three times with 75% ethanol. Samples were then let to air dry at room temperature for 10 minutes, resuspended in RNAse-free water and stored at -80°C. RNA concentration and purity were determined using a NanoDrop ND-1000 spectrophotometer (Nanodrop Technologies, Wilmington, DE, USA) and RNA integrity was determined by the RNA integrity number (RIN) using an Agilent TM 2100 Bioanalyzer and a RNA 6000 Nano LabChip Kit (Agilent Technologies, Palo Alto, CA, USA). The RIN values varied from 5.8 to 8.1 (mean RIN = 7.1). Detailed information about the RNA concentration and purity measurements is provided in S2 Table.

### Sample labeling and microarray hybridization

Five micrograms of total RNA per subject were used to synthesize cDNA. cDNA synthesis and *in vitro* transcription reactions for biotin-labeled complement RNA (cRNA) were carried out using One-Cycle cDNA synthesis kit and the GeneChip IVT labeling kit (Affymetrix, Inc., Santa Clara, California, USA). The biotinylated cRNA was then cleaned up and fragmented and hybridization cocktails were prepared according to the manufacturer’s protocol. Subsequently, the samples were hybridized for 16 hours on the GeneChip Human Genome U 133 Plus 2.0 arrays (sequence clusters were created from the UniGene database (Build 159, January 25, 2003)) and scanned using a GeneChip Scanner 3000 (Affymetrix).

### Microarray analysis and bioinformatics

The cell intensity files (CEL) generated from images of the scanned arrays were analyzed with Expression Console software (v.1.1) to create probe set summarization (CHP) files, evaluate the success of the individual hybridizations and identify possible outliers in the data set. In the initial steps we checked for arrays that had values outside our set bounds. Arrays with over 40% present calls and background average below 40, and cRNA samples which had a 3’/5’-ratio ≤ 3 for housekeeping gene glyceraldehydes-3-phosphate dehydroxygenase (GAPDH) and ≤10 for housekeeping gene actin were included for further analysis. In the initial set-up we included 40 structures for microarray analysis; however, based on the post-array quality measurements we had to exclude 12 donors from our study. The level of degradation in the included arrays was further explored by expanding the calculation of the 3’/5’-ratio to all present probes in the arrays. To that end, the CEL files were imported into R (www.r-project.org) and a probe-level analysis was performed using an R-package of functions called “*affy*”, specifically developed for the analysis of Affymetrix arrays [42]. This analysis returned all-probes-based RNA degradation plots for all arrays. RNA integrity levels were comparable for all samples (data not shown).

The CEL files were then imported into Partek Genomics Suited (Partek Incorporated, St. Louis, MO, USA) using a RMA (Robust Multi-chip Average) method, that includes quantile normalization, log2 transformation and median polish of probe-level intensities. The subsequent analyses included all (54676) probe sets. The data discussed in this manuscript have been deposited in NCBI’s Gene Expression Omnibus [43] and are accessible through GEO Series accession number GSE49036 (http://www.ncbi.nlm.nih.gov/geo/query/acc.cgi?acc=GSE49036).

Microarray data of all subjects were first subjected to an unsupervised hierarchical cluster analysis (Euclidian distance, average linkage) to explore the overlap in gene expression patterns among groups without pre-grouping the samples based upon clinical or pathological characteristics. Unsupervised hierarchical clustering analysis did not support any relation between SN gene expression changes and age, gender, post-mortem delay (PMD) or scan date of the included subjects. Moreover, no significant correlation between the 3’/5’-ratio of the two housekeeping genes GAPDH and actin and the pH of cerebrospinal fluid was observed (data not shown).

An analysis of covariance (ANCOVA) algorithm (with RIN as covariate to control for the variation caused by partial degradation of RNA) was used to compare probe set expression profiles between groups. The annotation of the probe sets is based on the annotation file (Release number 29, July 2009) and library files from Affymetrix.

To identify gene expression profiles differentially expressed during the progression of PD pathology, gene expression was compared between 1) controls, 2) Braak alpha-synuclein 1–2, 3) Braak alpha-synuclein 3–4, 4) Braak alpha-synuclein 5–6. Lists of probe sets were generated from the ANCOVAs using a Benjamini and Hochberg false discovery rate for multiple correction (BH-FDR) of 0.05 [44]. The lists of probe sets with significantly altered expression levels based on the uncorrected p-values in the separate groups compared to controls were used to investigate pathway alterations in the premotor stage and progression of PD pathology.

### Pathway analysis

First, the ranked probes (with uncorrected p-value <0.05) generated with ANCOVAs from the different groups (Braak alpha-synuclein 1–2, Braak alpha-synuclein 3–4 and Braak alpha-synuclein 5–6) compared to controls were imported into the software package Ingenuity Pathways Analysis version 8.8 (IPA; Ingenuity Systems, www.ingenuity.com) to assign pathway affiliation. Direct and indirect relationships were included in the analysis. Genes were assigned molecular pathways based on their GO annotation and Entrez Gene information (http://www.ncbi.nlm.nih.gov/gene). The p-value is determined by the probability that the association between the genes in the dataset and the canonical pathway is explained by chance alone. The significance value for pathway over-representation was calculated using Benjamini-Hochberg false discovery rate, to correct for multiple testing (BH-FDR) [44]. A BH-FDR <0.05 was considered significant.

### Validation

#### Quantitative PCR

Using quantitative PCR (qPCR), the expression levels of selected genes were studied in SN homogenates of 4 controls, 1 Braak alpha-synuclein stage 1–2, 4 Braak alpha-synuclein stage 3–4 and 4 alpha-synuclein stage 5–6 for technical validation. Subjects who were included from the microarray study which are listed in S1 Table. Six genes were chosen among the ones with significance (p-value ≤ 0.001) in the PD patients or the iLBD group compared to the control group: eukaryotic translation initiation factor 4A2 (EIF4A2), synaptophysin-like 1 (SYPL1), heat shock 70kDa protein 8 (HSPA8), lysosomal-associated membrane protein 2 (LAMP2), glutamate receptor, metabotropic 3 (GRM3) and fatty acid binding protein 7, brain (FABP7). The housekeeping genes ribosomal protein L27 (RPL27) and ribosomal protein S13 (RPS13) were selected from the list of housekeeping genes as these genes had the most stable expression profiles among all groups in the SN based on the microarray analyses. For reverse transcription of total RNA from SN homogenates, iScriptTM cDNA Synthesis Kit (BioRad Laboratories, Hercules, CA, USA) was used. The primers for the TaqMan assays were designed in the same region as the affymetrix probe set, spanning genomic intron-exon junctions, aiming to regions containing introns and as close as possible to the 3’ end of the sequence. Forward and reverse primer sequences were checked for possible secondary structures and dimer formation with the primer analysis tool available at OligoFaktory web portal (http://164.15.232.115/oligofaktory/index.jsp?tool=analysis) and at http://www.eurofinsgenomics.eu. All primers were purchased from Eurogentec S.A. (Liege, Belgium), and suitable probes were selected from the Human Universal Probe Library (Roche Applied Science, Mannheim, Germany). All PCR reactions were prepared with TaqMan Fast Universal PCR Master Mix (Applied Biosystems, 11 Norwalk, CT) and performed using a StepOne Plus Real-Time PCR instrument (Applied Biosystems). Per sample, three replicates and one negative control (RT-) were run in each experiment. Fifty nanograms of cDNA were used in each 20 microliters reaction. All reactions included a holding stage (15 minutes at 95°C) and 40 amplification cycles. Sequences of the primers and details of the each cycle are listed in S3 Table. Standard curves were generated for each assay run. The relative expression ratio of each target gene was calculated using the efficiency-corrected delta-delta Cq method [45].

The results of the quantitative PCR and normalized microarray expression were statistically analysed using a Pearson correlation coefficient. A p-value <0.05 was considered significant.

#### In situ hybridization

Using *in situ* hybridization, localization of the mRNA transcript was studied in unfixed frozen SN sections of PD, iLBD and control subjects. RNA probes for HSPA8, EIF4A2 and LAMP2 were generated using cDNA synthesized from total human brain RNA (iScriptTM cDNA Synthesis Kit, BioRad Laboratories, Hercules, CA, USA). A PCR amplification was performed with Phusion High-fidelity PCR kit (# F553L, Thermo Scientific) using primers containing T3 (sense) or T7 (antisense) promoters (sequences; S4 Table). Digoxigenin labeled RNA probes were generated by linear amplification using Maxiscript T7/T3 kit (#AM1322, Ambion) and DIG RNA labeling mix containing digoxigenin-UTP (#11277073910, Roche). Probes were checked for size on 1% agarose gel, and sequenced using an ABI3730 DNA analyzer (Applied Biosystems).

Unfixed frozen SN of PD was sectioned (40 μm) with a cryostat at -18°C, mounted on Superfrost Plus slides (Thermo Scientific) and stored at -80°C. Before the hybridization, slides were kept at room temperature for 30 minutes, fixed with 4% buffered PFA for 20 minutes, then washed with PBS and acetylated with 0.25% acetic anhydride in triethanolamine buffer for 10 minutes. After ethanol dehydration and chloroform treatment, sections were rehydrated and washed with PBS, followed by 2x saline sodium citrate buffer (SSC buffer). The probes were added to the hybridization mix (containing 50% formamide, 4x SSC, tRNA, 50x Denhards reagent and 10% dextran). The hybridization was performed overnight at 60°C. Post-hybridization washes were carried out with 1xSSC in a 60°C water bath and at room temperature, and with 2xSSC containing 0.3 units/ml Rnase A (Roche) in a 37°C water bath. Slides were then pre-incubated for 1 hour with a blocking solution containing 1% blocking powder (DIG nucleid acid detection kit, # 11 175 041 910, Roche) and subsequently incubated overnight at 4°C in humid chamber with anti-DIG AP Fab fragments (#11093274910, Roche) diluted 1:1500 in blocking solution. After buffer washings, the alkaline phosphatase colour reaction was started, incubating the slides with NBT/BCIP (diluted 1:50; #11681451001, Roche) at room temperature in humid chamber. The reaction was stopped in a stop buffer containing 1mM EDTA. Slides were then air dried and coverslipped with Merckoglas (Merck, New Jersey, USA).

## Results

### Clustering of gene expression profiles

**Fig 1:**
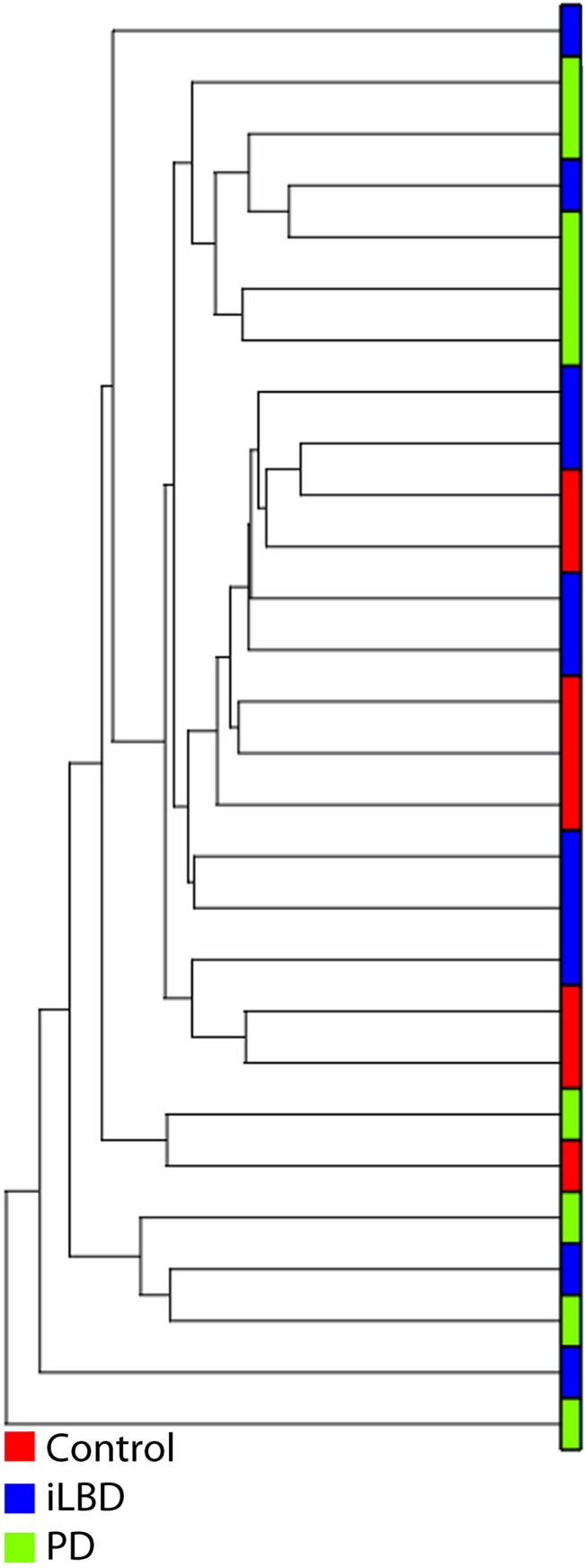
Unbiased hierarchical clustering of all gene expression profiles of iLBD, PD and control donors. Red is control, blue is iLBD and green is PD donor. The two main clusters are formed by 1) controls and iLBD and 2) PD and iLBD, indicating that the expression of iLBD is intermediate between control and PD. Seven samples clustered separately from the two main clusters. There were no technical reasons however, to exclude these donors from the analysis.

In order to identify differences in genome wide gene expression patterns between PD, iLBD and controls, we performed microarray analysis of the SN. First, an unbiased hierarchical cluster analysis was applied to the microarray dataset to study the clustering of the gene expression data of the 3 pathological groups (Fig 1). Within the cluster, there was full separation between PD and control samples, indicating substantial differences in gene expression profiles. The expression profiles of the iLBD subjects clustered either together with the control cases, or with the PD, but they didn’t form their own cluster, indicating that their expression levels are intermediate between control and PD gene expression profiles. Unexpectedly, seven samples clustered separately from the two main clusters. There were no technical reasons however, to exclude these donors from the analysis.

### Altered pathways in the SN in different stages of pathological progression

The ANCOVA of the four groups resulted in the identification of 137 probe sets (BH-FDR 0.05). Pathway analysis showed that 23 pathways were altered among all groups (8475 probe sets, unadjusted p<0.05). Pathways with alterations throughout disease progression in PD included EIF2 signaling (BH-FDR = 0.00000047), breast cancer regulation by Stathim 1 (BH-FDR = 0.000051), regulation of eIF4 and p70S6K signaling (BH-FDR = 0.00014), dopamine-DARPP32 feedback in cAMP signaling (BH-FDR = 0.00018) and mTOR signaling (BH-FDR = 0.00019). These pathways are involved in protein translation shut down, immune response and axonal degeneration.

In addition to confirming the involvement of several previously described pathways in end-stage PD, our dataset revealed other molecular alterations, which parallel the progression of PD pathology. Differentially expressed probe sets with unadjusted p<0.05 between Braak alpha-synuclein stage 1–2 subjects and controls (4294 probe sets), resulted in 131 significantly altered probe sets between the transcriptome of the SN in Braak 1–2 and Braak 5–6 stages compared to controls, indicating that alterations of the same molecular mechanisms occur in iLBD and PD. The top deregulated pathways are involved in cellular proliferation and include molecular mechanisms of cancer (BH-FDR = 0.000019), colorectal cancer metastasis signaling (BH-FDR = 0.000074), gap junction signaling (BH-FDR = 0.000074) and actin cytoskeleton signaling (BH-FDR = 0.00010). In addition, we found previously identified pathways from other studies, which were not altered in PD in our study, to be altered between Braak alpha-synuclein stage 1–2 subjects and controls. These pathways include PDGF signaling (BH-FDR = 0.00012), estrogen-dependent breast cancer signaling (BH-FDR = 0.00068), JAK/Stat signaling (BH-FDR = 0.00055), IGF-1 signaling (BH-FDR = 0.00089), glucocorticoid receptor signaling (BH-FDR = 0.0017), VEGF signaling (BH-FDR = 0.0012) and BMP signaling pathway (BH-FDR = 0.0025), suggesting that these pathways are altered in the earliest stages of the disease.

**Fig 2:**
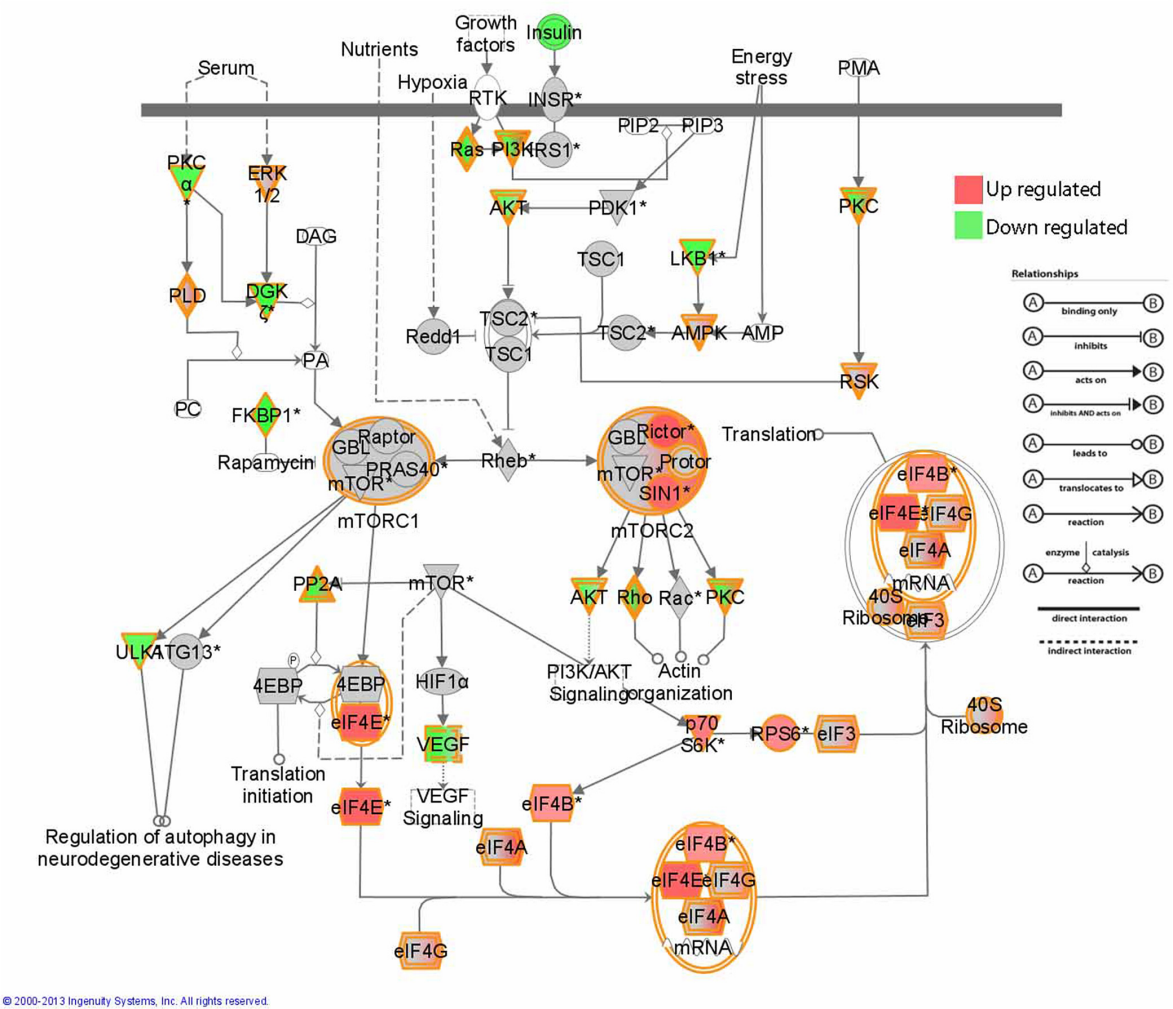
Alterations in elements of mTOR, EIF2 and EIF4 signaling pathways in Braak alpha-synuclein 1 and 2 compared to controls. Image generated using Ingenuity pathway analysis (IPA).

Between Braak alpha-synuclein stage 1–2 and Braak alpha-synuclein stage 3–4 (3084 probe sets), two pathways involved in immune response and protein translation were significantly altered: FLT3 signaling in progenitor cells (BH-FDR = 0.001) and EIF2 signaling (BH-FDR = 0.009). Finally, in Braak alpha-synuclein stage 5–6 compared to 3–4 (6602 probe sets), we observed 4 pathways to be altered, dopamine-DARPP32 feedback in cAMP signaling (BH-FDR = 0.0065), thrombin signaling (BH-FDR = 0.0065), cardiac beta-adrenergic signaling (BH-FDR = 0.0065) and CTLA4 signaling in cytotoxic L lymphocytes (BH-FDR = 0.0068). Several pathways deregulated during disease progression in PD are involved in macroautophagy: these include EIF2 signaling and regulation of eIF4 and p70S6K signaling and mTOR signaling. The alterations of the elements of these pathways in Braak alpha-synuclein stage 1–2 compared to controls are displayed in Fig 2. All pathways altered in Braak alpha-synuclein stage 1–2 compared to controls, Braak 3–4 compared to Braak 1–2, Braak 5–6 compared to Braak 3–4, and all the Braak groups compared to controls, are listed in S5 Table.

### Gene expression changes related to immune response, axonal degeneration and endocytosis in the Braak alpha-synuclein stages 1–2

From the analyses described above, we learned that the most pronounced changes in gene expression occur in pathways related to inflammation and immune response, axonal degeneration and macroautophagy in early stage PD. With regard to inflammation and immune response, several pathways were altered in the Braak alpha-synuclein stages 1–2. These include HMGB1 signaling (BH-FDR = 0.00015), which is a mediator of inflammation, CXCR2 signaling (BH-FDR = 0.00054) and P2Y purigenic receptor signaling (BH-FDR = 0.00056), which both are thought to mediate inflammation by cytokine signaling. Also GM-CSF signaling, which is considered to be part of the immune/inflammation cascade (BH-FDR = 0.00058) is altered. Other pathways found in our dataset related to inflammation and immune response include thrombin signaling (BH-FDR = 0.0012), B cell receptor signaling (BH-FDR = 0.00051) and T cell receptor signaling (BH-FDR = 0.0035). In addition, CTLA4 signaling in cytotoxic T lymphocytes was up regulated in Braak alpha-synuclein stage 1 and 2 (BH-FDR = 0.0038). The MHC II class receptors were also up regulated, indicating a disturbance of the extracellular matrix. When we looked at altered probe sets, an up regulation in HLA-DQA1 (p = 0.02, FC = 2.26) and HLA-DRA (p = 0.02, FC = 2.66) and HLA-DRB1 (p = 0.02, FC = 1.92) was observed in Braak alpha-synuclein stage 1–2 compared to controls, but not in other stages of the pathological progression. In addition to immune activation, we observed changes in pathways linked to inflammation, such as Interleukin (IL)-2,3,4,8 and 14 signaling pathways in Braak alpha-synuclein stage 1–2 (BH-FDR ranged between 0.001 and 0.09; see S5 Table).

In Braak alpha-synuclein stage 1–2, we observed a down regulation of pathways involved in cytoskeletal maintenance such as axonal guidance signaling (BH-FDR = 0.00010), gap junction signaling (BH-FDR = 0.000074), actin cytoskeleton signaling (BH-FDR = 0.00010), RhoGDI signaling (BH-FDR = 0.00056), protein kinase A signaling (BH-FDR = 0.0028) and regulation of actin-based motility by Rho (BH-FDR = 0.01). In the SN of donors with Braak alpha-synuclein stage 1–2, deregulated pathways related to dopamine functioning at the level of the synapse include dopamine-DARPP32 feedback in cAMP signaling (BH-FDR = 0.000093). We observed no difference in expression level of alpha-synuclein (p = 0.67) and the interactors of alpha-synuclein between Braak alpha-synuclein 1–2 compared to controls, but significant changes were observed for alpha-synuclein (p = 0.001) and most of the interactors in PD compared to controls (Table 2). When we focus on the anterograde transporter proteins, we observed a down regulation of kinesin light chain (KLC) (p = 0.02, FC = -1.42) and kinesin family 20A (KIF20A) (p = 0.02, FC = -1.17) in Braak alpha-synuclein stage 1–2 compared to controls. Among the pathways related to endocytosis, endocytosis mediated by clathrin (BH-FDR = 0.000010) was down regulated and virus entry through endocytic pathways (BH-FDR = 0.0079) was up regulated in Braak alpha-synuclein stage 1–2 compared to controls. In addition, markers for endosomes, such as transferrin (TF), early endosome antigen1 (EEA1) and member Ras oncogene family 4 (RAB4), were increased in subjects with Braak alpha-synuclein 1–2 compared to controls (Table 3), but not in PD compared to controls.

**Table 2:** Alterations in expression levels of alpha-synuclein and known interactors of alpha-synuclein in Braak 1–2 and PD compared to controls.

	Braak alpha-synuclein 1–2 compared to control		Braak alpha-synuclein 5–6 compared to control	
Gene name	p-value	Fold change	p-value	Fold change
Synaptotagmin II	7.5*10^−5^	1.92	0.14	1.19
Synaptophysin	4.3*10^−4^	2.35	4.3*10^−4^	2.05
Synapsin 3	1.8*10^−5^	1.45	0.89	1.02
Synphilin 1	0.03	-1.19	0.01	-1.2
Complexin 2	0.04	-1.12	8.7*10^−4^	-1.18
Synapsin 2	0.04	1.76	0.41	-1.2
Pra 1	0.13	1.26	0.19	-1.18
Syntaxin 1	0.15	-1.21	3.0*10^−5^	-1.76
GLUr1	0.22	-1.45	3.3*10^−3^	-2.23
Sptbn1	0.27	-1.30	0.03	1.59
Vamp2	0.39	-1.22	2.9*10^−3^	-1.91
Complexin 1	0.52	1.19	0.19	-1.36
Tubulin	0.56	-1.17	3.4*10^−3^	-2.05
VMAT2	0.67	-1.40	0.03	-4.6
Snap25	0.79	1.07	0.03	-1.66
Synapsin 1	0.82	-1.08	2.9*10^−3^	-2.43
Dynamin 1	0.92	-1.04	0.01	-2.44
alpha-synuclein	0.68	-1.12	9.6*10^−4^	-2.34

**Table 3:** Alterations in expression levels of genes involved in endocytosis in the early Braak alpha-synuclein stages 1 and 2 and PD, compared to controls

		Braak alpha-synuclein 1–2 compared to controls		Braak alpha-synuclein 5–6 compared to controls	
Gene	Gene Symbol	p-value	Fold-Change	p-value	Fold-Change
RAB4A, member RAS oncogene family	RAB4A	0.01	1.68	0.49	1.12
RAB5A, member RAS oncogene family	RAB5A	0.02	2.28	0.13	1.51
Ankyrin 2, neuronal	ANK2	0.01	2.11	0.20	1.33
Transferrin	TF	0.003	2.14	0.12	1.31
Early endosome antigen 1	EEA1	0.02	1.32	0.07	1.19

### Molecular pathways involved in end-stage PD compared to control in the SN

In total, 2657 probe sets, of which 976 were up regulated, and 1681 down regulated (BH-FDR <0.05), were altered in PD patients with Braak alpha-synuclein 5–6 compared to controls. The altered probe sets which displayed a fold change of ±1.50 or more are shown in S6 Table. Ingenuity Pathway analysis of differentially expressed genes at Braak stage 5–6 (12199 probe sets, ANCOVA unadjusted p<0.05) revealed 47 deregulated pathways (BH-FDR<0.05), which included PI3K/AKT signaling (BH-FDR = 0.00032), signaling by Rho family GTPases (BH-FDR = 0.00032), and EIF2 signaling (BH-FDR = 0.00032).

The following pathways were deregulated in SN tissue of PD patients with Braak alpha-synuclein stage 5–6 (BH-FDR<0.05) and have been previously described by others [19, 22, 24, 25, 28, 30, 33]: PI3K/AKT signaling (BH-FDR = 0.00032), PTEN signaling (BH-FDR = 0.00096), B cell receptor signaling (BH-FDR = 0.0019), mTOR signaling (BH-FDR = 0.0053), dopamine receptor signaling (BH-FDR = 0.03), ephrin receptor signaling (BH-FDR = 0.03), Huntington’s disease signaling (BH-FDR = 0.02), and axonal guidance signaling (BH-FDR = 0.02). Other previously identified pathways, such as estrogen receptor signaling and IGF-1 signaling, were not altered in our PD group. Elstner et al. [33] demonstrated that the differences in tissue processing and data generation can be overcome by focusing on pathway alterations. Here, we observed a large overlap between the pathways deregulated in end-stage PD and previously described datasets by others [19–29, 30, 33], which indicates that the observed changes in gene expression profiles in the SN post-mortem tissue are similar to those of other cohorts, even though different microarrays platforms were used. In Table 4, an overview of novel and previously identified deregulated pathways in end-stage PD is given. Novel pathways included signaling by rho family GTPases (BH-FDR = 0.00032), RhoGDI signaling (BH-FDR = 0.00072) and protein kinase A signaling (BH-FDR = 0.0026).

**Table 4:** Molecular pathways associated with the up- or down-regulated genes in end-stage PD (Braak 5–6) versus controls in our study, compared to other transcriptome studies.

IPA pathway category	PD compared to control**	Lesnick et al. 2007 [30] ***	Moran et al. 2006 [24]-medial SN***	Moran et al. 2006 [24]- lateral SN***	Zhang et al 2005 [28] ***	Hauser et al. 2005 [22] ***	Elstner et al. 2011 [33] ***	Simunovic et al. 2009 [25] ***	Bossers et al. 2009 [19] ***
EIF2 signaling	3.16*10^−4^						1.10*10^−3^	4.0*10^−4^	
PI3K/AKT signaling	3.16*10^−4^	0.01			0.02		0.03	1.8*10^−3^	
Signaling by Rho Family GTPases	3.16*10^−4^ *								
RhoGDI signaling	7.20*10^−4^ *								
PTEN signaling	9.55*10^−4^	0.01	0.02	0.03					0.03
Regulation of Stathmin/Breast Cancer Regulation by Stathmin1	1.29*10^−3^						8.9*10^−5^	7.8*10^−4^	
B cell receptor signaling	1.86*10^−3^		0.01		1.3*10^−3^				
Role of NFAT in cardiac hypertrophy	2.57*10^–3^								0.02
Protein kinase A signaling	2.60*10^−3^ *								
Rac signaling	2.69*10^−3^						0.01	0.02	
Phospholipase C signaling	4.10*10^−3^ *								
mTOR signaling	5.25*10^−3^						0.02	0.02	
Regulation of eIF4 and p70S6K signaling	5.25*10^−3^						4.8*10^−5^	1.5*10^−4^	
Fcgamma Receptor-mediated phagocytosis in macrophages and monocytes	5.25*10^−3^ ^*^								
RhoA signaling	0.013*								
CXCR4 signaling	0.015						0.02	0.02	0.02
Tight junction signaling	0.018*								
NGF signaling	0.018*								
Gap Junction signaling	0.018*								
Axonal guidance signaling	0.018	0.05		0.01			0.02	8.5*10^−4^	
Huntington’s disease signaling	0.019		9.1*10^−4^	4.8*10^−3^		0.02	0.01	1.5*10^−3^	0.00
Cellular effects of Sildenafil (Viagra)	0.019*								
Galpha12/13 signaling	0.019								0.03
p70S6K signaling	0.019						0.01	9.1*10^−5^	
Virus entry via endocytic pathways	0.019						0.04	2.5*10^−4^	
G beta gamma Signaling	0.027*								
Ephrin receptor signaling	0.028				0.03		2.5*10^−3^		
Molecular mechanisms of cancer	0.028*								
CTLA4 signaling in Cytotoxic T lymphocytes	0.028*								
Regulation of IL-2 expression in activated and anergic T Lymphocytes	0.028*								
GNRH signaling	0.030								3.2*10^−3^
Role of NFAT in regulation of the immune response	0.030*								
SAPK/JNK signaling	0.030*								
Telomerase signaling	0.030*								
CDK5 signaling	0.030*								
Ceramide signaling	0.030*								
Chronic myeloid leukemia signaling	0.030*								
PI3K signaling in B lymphocytes	0.030*								
Dopamine receptor signaling/Dopamine DARPP feedback in cAMP signaling	0.030	0.03	0.01	3.0*10^−3^	0.01	0.01			
Germ cell-sertoli cell junction signaling	0.035						0.03	2.2*10^−3^	
Reelin signaling in neurons	0.035						3.6*10^−3^	0.01	
ILK signaling	0.036						0.01	0.02	
Thrombin signaling	0.045						0.03	2.5*10^−3^	
Alpha-adrenergic Signaling	0.045								0.01
ERK/MAPK Signaling	0.046*								
HGF Signaling	0.048*								

* novel pathways in the SN in PD compared to control

** BH-FDR<0.05 displayed;

***Fisher p-value<0.05 displayed

### Validation

#### Correlation between microarray expression and qPCR expression data

We found a significant correlation between the microarray expression and the normalized gene expression calculated using Pfaffl for LAMP2 (r = 0.71, p<0.01), GRM3 (r = 0.81, p = 0.01), FABP7 (r = 0.84, p = 0.01), HSPA8 (r = 0.54, p = 0.049), EIF4A2 (r = 0.63, p = 0.02) and SYPL1 (r = 0.61, p = 0.02). Plots of the qPCR per pathological group are shown in S1 Fig.

#### In situ hybridization shows expression in glial and neuronal cells

LAMP2 expression was detected in neuronal cells in PD SN, but not in control and iLBD. EIF4A2 showed moderate expression in dopaminergic neurons in control and iLBD cases, and lower expression in PD donors. HSPA8 showed strong expression in the dopaminergic neurons in all groups; in PD donors a lower number of positive neurons was clearly detectable (S2 Fig).

## Discussion

**Fig 3:**
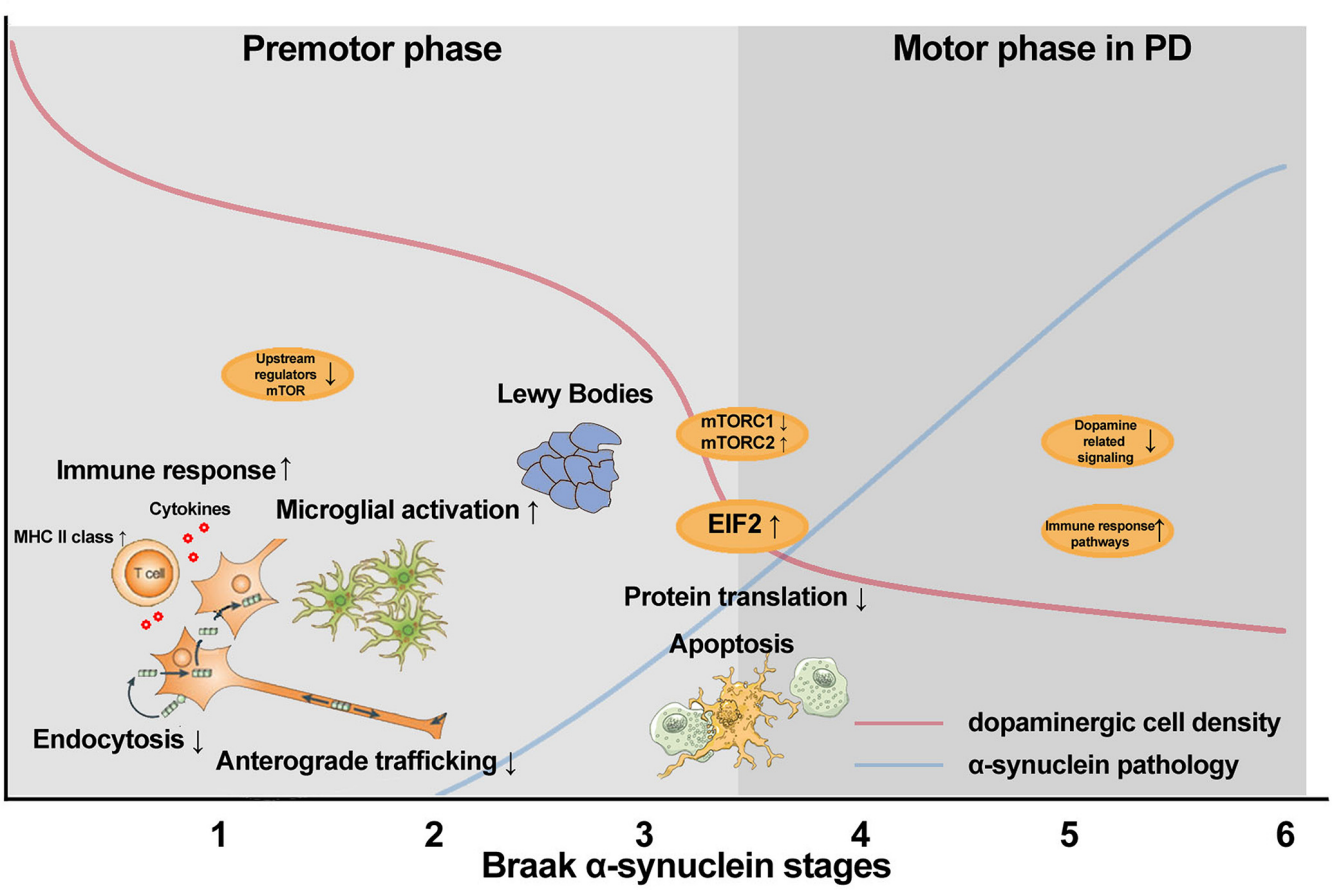
Schematic overview of molecular processes altered during disease progression in the SN of PD, identified using transcriptome analysis. In Braak alpha-synuclein 1 and 2, minimal cell loss is observed along with decreased endocytosis and anterograde trafficking, and increased immune response and microglial activation. In addition, we observed down regulation of mRNA levels of the upstream regulators in mTOR pathway. In Braak alpha-synuclein 3 and 4, a steep decline in cell loss and increase in alpha-synuclein pathology is observed together disturbed regulation of protein production and apoptosis of the nigral dopaminergic cells. In Braak alpha-synuclein 5 and 6 compared to controls, severe cell loss and alpha-synuclein aggregation is observed [41]. At this advanced stage, alterations in pathways related to dopaminergic signaling and immune response are still observed.

In summary, the transcriptome of post-mortem SN tissue of donors with Braak alpha-synuclein stages 0 to 6 revealed a consistent deregulation of pathways related to macroautophagy and protein synthesis, including EIF2 signaling, mTOR signaling and regulation of eIF4 and p70S6K signaling during the progression of PD pathology. When focusing specifically on Braak alpha-synuclein 1–2 compared to controls, the transcriptome analysis revealed changes in pathways linked to immune response and axonal degeneration, including B cell receptor signaling, protein kinase A signaling and axonal guidance signaling. In addition, in Braak alpha-synuclein 1–2 stages, we observed a down regulation of clathrin-mediated endocytosis. These data indicate that endocytosis, inflammation and axonal function are compromised in the early stages of PD, prior to the presence of local alpha-synuclein pathology in the SN [5, 6], whereas EIF2 and mTOR signaling is deregulated on mRNA expression level throughout disease progression in PD. A summary of the main transcriptional events and local pathology in the SN during disease progression in PD is given in Fig 3.

### Deregulation of mTOR and EIF2 signaling pathways during disease progression in PD

Several pathways were deregulated on mRNA expression level in the early pathological stages and remained altered in the later stages of PD in the SN, such as mTOR signaling, EIF2 signaling, and regulation of eIF4 and p70S6K signaling. mTOR and EIF2/EIF4 have previously been linked to PD in post-mortem transcriptome studies [25, 34], and mutations in the translation initiator EIF4G1 have recently been identified in familial PD [46]. mTOR consist of two different sub-pathways; 1) the mTOR1 pathway results in the transient shutdown of protein translation through phosphorylation of the alpha-subunit of eukaryotic translation initiation factor, EIF2, which showed up regulated levels of mRNA in our dataset. Rapamycin has been used in clinical trials [47, 48] and inhibits the activity of mTOR1 complex. This results in an induction of macroautophagy, as demonstrated in a mouse model for PD [49]; 2) The other sub-pathway, mTOR2, promotes apoptosis and was up regulated in our dataset, suggested that the cells might be degenerating. These pathways are part of a network that relays metabolic signals to adaptive changes in protein translation and gene expression [50, 51]. As these pathways are consistently deregulated in our dataset, even before the onset of motor symptoms, they might contain biomarker candidates that may aid in diagnosing PD at an earlier stage. The *in situ* hybridization showed that the expression of an important component in these pathways, EIF4A2, is expressed in neuromelanin containing neurons. This indicates that the alterations in mTOR and EIF2 signaling represent alterations in neuronal pathways prior to the formation of aggregates in the SN. Moreover, a recent transcriptome study from Mutez and colleagues showed that deregulation of the EIF2 signaling pathway is evident in samples of peripheral blood mononuclear cells of genetic as well as sporadic PD patients compared age-matched healthy controls [52] providing further evidence for a key role of this pathway in both genetic and sporadic forms of PD.

### Involvement of immune response during the progression of PD

Recently, it has been shown that mutations in the non-coding regions of HLA-DR are associated with familial PD or increase the risk of developing PD [53]. In our dataset, we also observed an up regulation of several HLA genes in the early pathological stages, but not in the later stages. We demonstrated that GM-CSF signaling, thrombin signaling, B cell receptor signaling, CTLA4 signaling in cytotoxic T lymphocytes and T cell receptor signaling were altered. These findings point towards a key role for deficient immune responses in the early stages of PD, where limited cell loss is present. Alterations in these pathways might be triggered by oxidative stress or abnormal forms of alpha-synuclein (reviewed in [54]). In later stages 5 and 6, we observed only up regulated B cell receptor signaling. As there is a large decrease in dopaminergic cells in end-stage PD, the up regulation in immune response related pathways might be an effect of the increased glial to neurons ratio observed in the SN tissue during disease progression.

### Macroautophagy and axonal dysfunction in the early stages of PD pathology

In PD, there is an accumulation of autophagosomes, and mitochondria and microtubule-directed traffic might be the main players in the regulation of macroautophagy in PD [55]. In Braak alpha-synuclein stage 1–2, we observed an up regulation of genes related to endosomes, suggesting accumulation of endosomes, possibly including autophagosomes. In addition, pathways related to endocytosis were deregulated, as a probable protection mechanism against further accumulation of endosomes. LAMP2 is a lysosomal protein and involved in chaperone-mediated autophagy (CMA) [56]. We observed an up regulation in LAMP2 expression with increasing Braak stages, which we confirmed using qPCR, and *in situ* hybridization showed that there is a strong expression of LAMP2 in glial cells in PD. These data and the regulation of mTOR in the early stages of PD pathology suggest that macroautophagy and CMA are deregulated in the early stages, and might play a role in alpha-synuclein aggregation in the SN in elderly. No alterations in mitochondrial dysfunction have been identified in the transcriptome of Braak alpha-synuclein 1–2 donors, which indicates that mitochondrial dysfunction might be a later event in the pathogenesis of PD.

The most significant deregulated pathways in Braak alpha-synuclein stages 1–2 are involved in cytoskeleton stability and maintenance. We observed that axonal guidance signaling, actin cytoskeleton signaling and regulation of actin-based motility by Rho are down regulated in the early stages of PD pathology. This suggests that axonal dysfunction might contribute to the cell death cascade, as limited dopaminergic loss has been observed in these stages [10, 41]. Our data confirm that synaptic dysfunction is involved in the pathogenesis of PD [34] and support the “dying-back” hypothesis, which postulates an initial dysfunction at the level of the synapse and/or axonal transport disruption, prior to neuronal loss.

Changes in alpha-synuclein mRNA levels were subtle, and due to the low levels of endogenous alpha-synuclein mRNA expression and large biological variability, it might not be possible to observe alterations in transcriptome levels of alpha-synuclein and its interactors in the early Braak stages in a relatively small sample size. When we focused on PD compared to controls, we observed a significant decline in transcriptome levels of alpha-synuclein and its interactors such as vesicular monoamine transporter 2 (VMAT2), synapsin 1, dynamin 1 and tubulin, which is in line with other studies [34]. An explanation for the axonal dysfunction might be that the misfolded state of alpha-synuclein disrupts anterograde transport of proteins to the synapse [57, 58]. We observed a decrease in the anterograde transporters (KHC and KLC) in Braak alpha-synuclein 1–2, whereas in advanced PD (Braak alpha-synuclein 5–6) dynein, a retrograde transporter, was affected. We were able to find similar changes in axonal transport motor proteins as Chu et al. [59], who investigated known axonal transport proteins, such as kinesins and dynein, in a rat model for over expression of alpha-synuclein. This may implicate that even though mRNA levels of alpha-synuclein are not changing in Braak alpha-synuclein 1–2 compared to controls, the transport of alpha-synuclein to the synapse might be disturbed.

To summarize, the deregulated pathways indicate that axonal guidance signaling is compromised. No alterations in mRNA levels of alpha-synuclein and its interactors were observed in Braak stages 1–2, but a disturbance in anterograde trafficking might result in reduced synaptic functioning.

To conclude, transcriptome analysis revealed that mTOR and EIF2 signaling are deregulated in the SN throughout the progression of PD pathology. Prior to the presence of alpha-synuclein aggregates, molecular mechanisms involved in axonal dysfunction, immune response and disturbed endocytosis may play a role in the neuronal cell death and the formation of protein aggregates in PD. Specific elements of these pathways and cellular processes may hold the key to altering the disease progression in PD.

## Supporting Information

S1 Table Detailed clinical and pathological characteristics of donors included in the study. (XLS)

S2 Table RNA quality data. Per sample, the RNA concentration, purity measurements and RIN value are displayed.
(XLS)

S3 Table Targets for qPCR and the properties of the created primers (XLS)

S4 Table Primers for *in situ* hybridization. Hybridization temperature for all primers was 60°C
(XLS)

S5 Table Altered molecular pathways based on 4 groups (control, Braak 1–2, Braak 3–4, and Braak 5–6) analysis: comparisons among all consecutive pathological groups, and between all pathological groups and controls (B-H p-value displayed). Data in bold indicate p>0.01.
(XLS)

S6 Table Genes with altered expression level in the SN of PD donors compared to controls (BH-FDR<0.05, Fold change ±1.50) (XLS)

S1 Fig Plots of qPCR data based on the clinical and pathological diagnosis of the donors. Data on LAMP2, GRM3, FABP7, HSPA8, EIF4A2 and SYPL1 are shown for controls, iLBD (Braak 1–3) and PD (Braak 4–6).
(TIF)

S2 Fig *In situ* hybridization of LAMP2, EIF4A2 and HSPA8 in the substantia nigra of non-demented control (A, D, G), iLBD (B, E, H) and PD (C, F, I) donors. LAMP2 expression (A, B, C) is detected in neuronal cells in PD substantia nigra. EIF4A2 (D, E, F) shows lower expression in the dopaminergic neurons of PD donors compared to iLBD and control cases; HSPA8 (G, H, I) shows strong expression in the dopaminergic neurons in all groups. Arrow heads indicate dopaminergic neurons.
(JPG)

## References

[1] Greffard S, Verny M, Bonnet AM, Beinis JY, Gallinari C, Meaume S et al Motor score of the Unified Parkinson Disease Rating Scale as a good predictor of Lewy body-associated neuronal loss in the substantia nigra. Arch Neurol 2006 4;63:584–588. 16606773 10.1001/archneur.63.4.584

[2] Spillantini MG, Schmidt ML, Lee VM, Trojanowski JQ, Jakes R, Goedert M. Alpha-synuclein in Lewy bodies. Nature 1997 8 28;388:839–840. 9278044 10.1038/42166

[3] Alafuzoff I, Ince PG, Arzberger T, Al-Sarraj S, Bell J, Bodi I et al Staging/typing of Lewy body related alpha-synuclein pathology: a study of the BrainNet Europe Consortium. Acta Neuropathol 2009 6;117:635–652. 10.1007/s00401-009-0523-2 19330340

[4] Braak H, Braak E. Neuropathological stageing of Alzheimer-related changes. Acta Neuropathol 1991;82:239–259. 1759558 10.1007/BF00308809

[5] Braak H, Del Tredici K, Rub U, De Vos RA, Jansen Steur EN, Braak E. Staging of brain pathology related to sporadic Parkinson’s disease. Neurobiol Aging 2003 3;24:197–211. 12498954 10.1016/s0197-4580(02)00065-9

[6] Braak H, Bohl JR, Muller CM, Rub U, De Vos RA, Del Tredici K. Stanley Fahn Lecture 2005: The staging procedure for the inclusion body pathology associated with sporadic Parkinson’s disease reconsidered. Mov Disord 2006 12;21:2042–2051. 17078043 10.1002/mds.21065

[7] Wolters EC, Braak H. Parkinson’s disease: premotor clinico-pathological correlations. J Neural Transm Suppl 2006;309–319. 17017546 10.1007/978-3-211-45295-0_47

[8] Hawkes CH, Del Tredici K, Braak H. A timeline for Parkinson’s disease. Parkinsonism Relat Disord 2010 2;16:79–84. 10.1016/j.parkreldis.2009.08.007 19846332

[9] DelleDonne A, Klos KJ, Fujishiro H, Ahmed Z, Parisi JE, Josephs KA, et al Incidental Lewy body disease and preclinical Parkinson disease. Arch Neurol 2008 8;65:1074–1080. 10.1001/archneur.65.8.1074 18695057

[10] Milber JM, Noorigian JV, Morley JF, Petrovitch H, White L, Ross GW, et al. Lewy pathology is not the first sign of degeneration in vulnerable neurons in Parkinson disease. Neurology 2012 Nov 14. 10.1212/WNL.0b013e318278fe32 PMC3578379 23152586

[11] Dickson DW, Uchikado H, Fujishiro H, Tsuboi Y. Evidence in favor of Braak staging of Parkinson’s disease. Mov Disord 2010;25 Suppl 1:S78–S82. 10.1002/mds.22637 20187227

[12] Halliday GM, McCann H. Human-based studies on alpha-synuclein deposition and relationship to Parkinson’s disease symptoms. Exp Neurol 2008 1;209:12–21. 17706644 10.1016/j.expneurol.2007.07.006

[13] Kingsbury AE, Bandopadhyay R, Silveira-Moriyama L, Ayling H, Kallis C, Sterlacci W, et al Brain stem pathology in Parkinson’s disease: an evaluation of the Braak staging model. Mov Disord 2010 11 15;25:2508–2515. 10.1002/mds.23305 20818670

[14] Zaccai J, Brayne C, McKeith I, Matthews F, Ince PG. Patterns and stages of alpha-synucleinopathy: Relevance in a population-based cohort. Neurology 2008 3 25;70:1042–1048. 10.1212/01.wnl.0000306697.48738.b6 18362284

[15] Sundal C, Fujioka S, Uitti RJ, Wszolek ZK. Autosomal dominant Parkinson’s disease. Parkinsonism Relat Disord 2012 1;18 Suppl 1:S7–10. 10.1016/S1353-8020(11)70005-0 22166459

[16] Simon-Sanchez J, van Hilten JJ, van de Warrenburg WB, Post B, Berendse HW, Arepelli S, et al Genome-wide association study confirms extant PD risk loci among the Dutch. Eur J Hum Genet 2011 6;19:655–661. 10.1038/ejhg.2010.254 21248740 PMC3110043

[17] Hamza TH, Zabetian CP, Tenesa A, Laederach A, Montinurro J, Yearout D, et al Common genetic variation in the HLA region is associated with late-onset sporadic Parkinson’s disease. Nat Genet 2010 9;42:781–785. 10.1038/ng.642 20711177 PMC2930111

[18] Rochet JC, Hay BA, Guo M. Molecular insights into Parkinson’s disease. Prog Mol Biol Transl Sci 2012;107:125–188. 10.1016/B978-0-12-385883-2.00011-4 22482450

[19] Bossers K, Meerhoff G, Balesar R, van Dongen JW, Kruse CG, Swaab DF, et al Analysis of gene expression in Parkinson’s disease: possible involvement of neurotrophic support and axon guidance in dopaminergic cell death. Brain Pathol 2009 1;19:91–107. 10.1111/j.1750-3639.2008.00171.x 18462474 PMC8094761

[20] Cantuti-Castelvetri I, Keller-McGandy C, Bouzou B, Asteris G, Clark TW, Frosch MP, et al Effects of gender on nigral gene expression and parkinson disease. Neurobiol Dis 2007 6;26:606–614. 17412603 10.1016/j.nbd.2007.02.009 PMC2435483

[21] Grunblatt E, Mandel S, Jacob-Hirsch J, Zeligson S, Amariglo N, Rechavei G, et al Gene expression profiling of parkinsonian substantia nigra pars compacta; alterations in ubiquitin-proteasome, heat shock protein, iron and oxidative stress regulated proteins, cell adhesion/cellular matrix and vesicle trafficking genes. J Neural Transm 2004 12;111:1543–1573. 15455214 10.1007/s00702-004-0212-1

[22] Hauser MA, Li YJ, Xu H, Noureddine MA, Shao YS, Gullans SR, et al Expression profiling of substantia nigra in Parkinson disease, progressive supranuclear palsy, and frontotemporal dementia with parkinsonism. Arch Neurol 2005 6;62:917–921. 15956162 10.1001/archneur.62.6.917

[23] Miller RM, Kiser GL, Kaysser-Kranich TM, Lockner RJ, Palaniappan C, Federoff HJ. Robust dysregulation of gene expression in substantia nigra and striatum in Parkinson’s disease. Neurobiol Dis 2006 2;21:305–313. 16143538 10.1016/j.nbd.2005.07.010

[24] Moran LB, Duke DC, Deprez M, Dexter DT, Pearce RK, Graeber MB. Whole genome expression profiling of the medial and lateral substantia nigra in Parkinson’s disease. Neurogenetics 2006 3;7:1–11. 16344956 10.1007/s10048-005-0020-2

[25] Simunovic F, Yi M, Wang Y, Macey L, Brown LT, Krichevsky AM, et al Gene expression profiling of substantia nigra dopamine neurons: further insights into Parkinson’s disease pathology. Brain 2009 7;132:1795–1809. 10.1093/brain/awn323 19052140 PMC2724914

[26] Sutherland GT, Matigian NA, Chalk AM, Anderson MJ, Silburn PA, Mackey-Sim A, et al A cross-study transcriptional analysis of Parkinson’s disease. PLoS One 2009;4:e4955 10.1371/journal.pone.0004955 19305504 PMC2654916

[27] Vogt IR, Lees AJ, Evert BO, Klockgether T, Bonin M, Wullner U. Transcriptional changes in multiple system atrophy and Parkinson’s disease putamen. Exp Neurol 2006 6;199:465–478. 16626704 10.1016/j.expneurol.2006.01.008

[28] Zhang Y, James M, Middleton FA, Davis RL. Transcriptional analysis of multiple brain regions in Parkinson’s disease supports the involvement of specific protein processing, energy metabolism, and signaling pathways, and suggests novel disease mechanisms. Am J Med Genet B Neuropsychiatr Genet 2005 8 5;137B:5–16. 15965975 10.1002/ajmg.b.30195

[29] Duke DC, Moran LB, Pearce RK, Graeber MB. The medial and lateral substantia nigra in Parkinson’s disease: mRNA profiles associated with higher brain tissue vulnerability. Neurogenetics 2007 4;8:83–94. 17211632 10.1007/s10048-006-0077-6

[30] Lesnick TG, Papapetropoulos S, Mash DC, Ffrench-Mullen J, Shehadeh L, de Andrade M, et al A genomic pathway approach to a complex disease: axon guidance and Parkinson disease. PLoS Genet 2007 6;3:e98 17571925 10.1371/journal.pgen.0030098 PMC1904362

[31] Botta-Orfila T, Sanchez-Pla A, Fernandez M, Carmona F, Ezquerra M, Tolosa E. Brain transcriptomic profiling in idiopathic and LRRK2-associated Parkinson’s disease. Brain Res 2012 7 23;1466:152–157. 10.1016/j.brainres.2012.05.036 22634372

[32] Botta-Orfila T, Tolosa E, Gelpi E, Sanchez-Pla A, Marti MJ, Valldeoriola F, et al Microarray expression analysis in idiopathic and LRRK2-associated Parkinson’s disease. Neurobiol Dis 2012 1;45:462–468. 10.1016/j.nbd.2011.08.033 21946334

[33] Elstner M, Morris CM, Heim K, Bender A, Mehta D, Jaros E, et al Expression analysis of dopaminergic neurons in Parkinson’s disease and aging links transcriptional dysregulation of energy metabolism to cell death. Acta Neuropathol 2011 7;122:75–86. 10.1007/s00401-011-0828-9 21541762

[34] Pienaar IS, Burn D, Morris C, Dexter D. Synaptic protein alterations in Parkinson’s disease. Mol Neurobiol 2012 2;45:126–143. 10.1007/s12035-011-8226-9 22205299

[35] Exner N, Lutz AK, Haass C, Winklhofer KF. Mitochondrial dysfunction in Parkinson’s disease: molecular mechanisms and pathophysiological consequences. EMBO J 2012 7 18;31:3038–3062. 10.1038/emboj.2012.170 22735187 PMC3400019

[36] Alafuzoff I, Arzberger T, Al-Sarraj S, Bodi I, Bogdanovic N, Braak H, et al Staging of neurofibrillary pathology in Alzheimer’s disease: a study of the BrainNet Europe Consortium. Brain Pathol 2008 10;18:484–496. 10.1111/j.1750-3639.2008.00147.x 18371174 PMC2659377

[37] Alafuzoff I, Thal DR, Arzberger T, Bogdanovic N, Al Sarraj S, Bodi I, et al Assessment of beta-amyloid deposits in human brain: a study of the BrainNet Europe Consortium. Acta Neuropathol 2009 3;117:309–320. 10.1007/s00401-009-0485-4 19184666 PMC2910889

[38] Jellinger KA. A critical evaluation of current staging of alpha-synuclein pathology in Lewy body disorders. Biochim Biophys Acta 2009 7;1792:730–740. 10.1016/j.bbadis.2008.07.006 18718530

[39] Frigerio R, Fujishiro H, Ahn TB, Josephs KA, Maraganore DM, DelleDonne A, et al Incidental Lewy body disease: do some cases represent a preclinical stage of dementia with Lewy bodies? Neurobiol Aging 2011 5;32:857–863. 10.1016/j.neurobiolaging.2009.05.019 19560232 PMC3366193

[40] Schmitz C, Hof PR. Design-based stereology in neuroscience. Neuroscience 2005;130:813–831. 15652981 10.1016/j.neuroscience.2004.08.050

[41] Dijkstra AA, Voorn P, Berendse HW, Groenewegen HJ, Netherlands Brain Bank, Rozemuller AJ, et al Stage-dependent nigral neuronal loss in incidental Lewy body and Parkinson’s disease. 2014. Mov Disord. 2014 9;29(10):1244–51. 10.1002/mds.25952 24996051

[42] Gautier L, Cope L, Bolstadt, BM, Irizarry, RA, affy—analysis of Affymetrix GeneChip data at the probe level. Bioinformatics, 2004 20(3): p. 307–15. 14960456 10.1093/bioinformatics/btg405

[43] Edgar R, Domrachev M, Lash AE. Gene Expression Omnibus: NCBI gene expression and hybridization array data repository. Nucleic Acids Res 2002 1 1;30:207–210. 11752295 10.1093/nar/30.1.207 PMC99122

[44] Benjamini Y, Hochberg Y. Controlling the false discovery rate: a practical and powerful approach to multiple testing. Journal of the Royal Statistical Society, 1995:289–300.

[45] Pfaffl MW. A new mathematical model for relative quantification in real-time RT-PCR. Nucleic Acids Res 2001 5 1;29:e45 11328886 10.1093/nar/29.9.e45 PMC55695

[46] Chartier-Harlin MC, Dachsel JC, Vilarino-Guell C, Lincoln SJ, Lepretre F, Hulihan MM, et al Translation initiator EIF4G1 mutations in familial Parkinson disease. Am J Hum Genet 2011 9 9;89:398–406. 10.1016/j.ajhg.2011.08.009 21907011 PMC3169825

[47] Rodriguez AE, Palacios I, Rodriguez-Granillo AM, Mieres JR, Tarragona S, Fernandez-Pereira C, et al Comparison of cost-effectiveness of oral rapamycin plus bare-metal stents versus first generation of drug-eluting stents (from the Randomized Oral Rapamycin in Argentina [ORAR] 3 trial). Am J Cardiol 2014 3 1;113:815–821. 10.1016/j.amjcard.2013.11.033 24528614

[48] Liesveld JL, O’Dwyer K, Walker A, Becker MW, Ifthikharuddin JJ, Mulford D, et al A phase I study of decitabine and rapamycin in relapsed/refractory AML. Leuk Res 2013 12;37:1622–1627. 10.1016/j.leukres.2013.09.002 24138944

[49] Liu K, Shi N, Sun Y, Zhang T, Sun X. Therapeutic effects of rapamycin on MPTP-induced Parkinsonism in mice. Neurochem Res 2013 1;38:201–207. 10.1007/s11064-012-0909-8 23117422

[50] Bishop NA, Lu T, Yankner BA. Neural mechanisms of ageing and cognitive decline. Nature 2010 3 25;464:529–535. 10.1038/nature08983 20336135 PMC2927852

[51] Hock MB, Kralli A. Transcriptional control of mitochondrial biogenesis and function. Annu Rev Physiol 2009;71:177–203. 10.1146/annurev.physiol.010908.163119 19575678

[52] Mutez E, Nkiliza A, Belarbi K, de Broucker A, Vanbesien-Maillot C, Bleuse S, et al Involvement of the immune system, endocytosis and EIF2 signaling in both genetically determined and sporadic forms of Parkinson’s disease. Neurobiol Dis 2014 3;63:165–170. 10.1016/j.nbd.2013.11.007 24269915

[53] Hamza TH, Zabetian CP, Tenesa A, Laederach A, Montimurro J, Yearot D, et al Common genetic variation in the HLA region is associated with late-onset sporadic Parkinson’s disease. Nat Genet 2010 9;42:781–785. 10.1038/ng.642 20711177 PMC2930111

[54] Hirsch EC, Hunot S. Neuroinflammation in Parkinson’s disease: a target for neuroprotection? Lancet Neurol 2009 4;8:382–397. 10.1016/S1474-4422(09)70062-6 19296921

[55] Arduino DM, Esteves AR, Cardoso SM. Mitochondria drive autophagy pathology via microtubule disassembly: a new hypothesis for Parkinson disease. Autophagy 2013 1;9:112–114. 10.4161/auto.22443 23075854 PMC3542213

[56] Orenstein SJ, Kuo SH, Tasset I, Arias E, Koga H, Fernandez-Caresa I et al Interplay of LRRK2 with chaperone-mediated autophagy. Nat Neurosci 2013 4;16:394–406. 10.1038/nn.3350 23455607 PMC3609872

[57] Saha AR, Hill J, Utton MA, Asuni AA, Ackerley S, Grierson AJ et al Parkinson’s disease alpha-synuclein mutations exhibit defective axonal transport in cultured neurons. J Cell Sci 2004 3 1;117:1017–1024. 14996933 10.1242/jcs.00967

[58] Yang ML, Hasadsri L, Woods WS, George JM. Dynamic transport and localization of alpha-synuclein in primary hippocampal neurons. Mol Neurodegener 2010;5:9 10.1186/1750-1326-5-9 20181133 PMC2830200

[59] Chu Y, Morfini GA, Langhamer LB, He Y, Brady ST, Kordower JH. Alterations in axonal transport motor proteins in sporadic and experimental Parkinson’s disease. Brain 2012 7;135:2058–2073. 10.1093/brain/aws133 22719003 PMC4571141

