# Supplementary figures and images for: Evidence for Immune Response, Axonal Dysfunction and Reduced Endocytosis in the Substantia Nigra in Early Stage Parkinson’s Disease
Source: PLoS One. 2015 Jun 18;10(6):e0128651. doi: 10.1371/journal.pone.0128651 (PMC4472235; doi:10.1371/journal.pone.0128651)

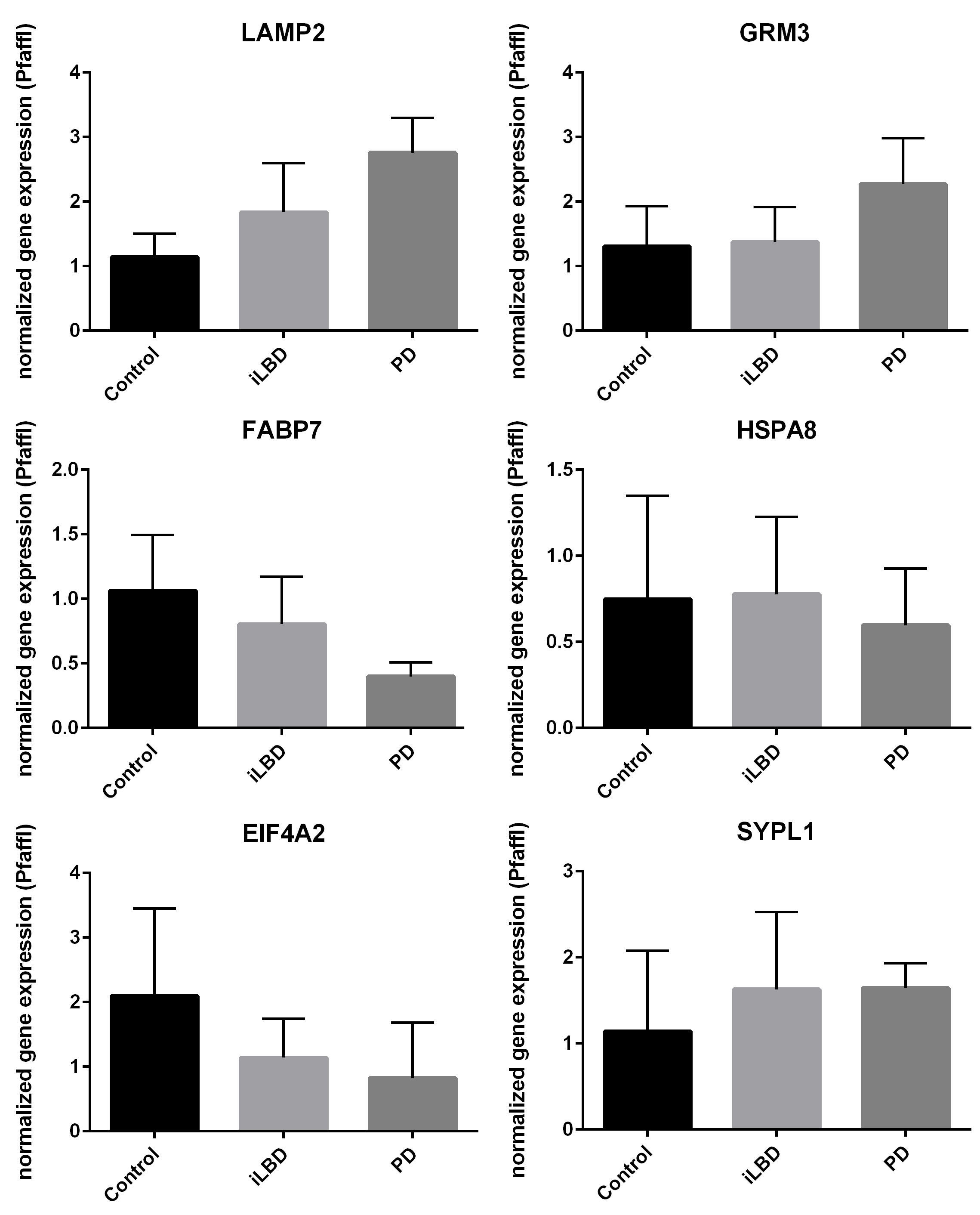

Supplement: S1 Fig — Data on LAMP2, GRM3, FABP7, HSPA8, EIF4A2 and SYPL1 are shown for controls, iLBD (Braak 1–3) and PD (Braak 4–6). (TIF) [file pone.0128651.s007.tif]

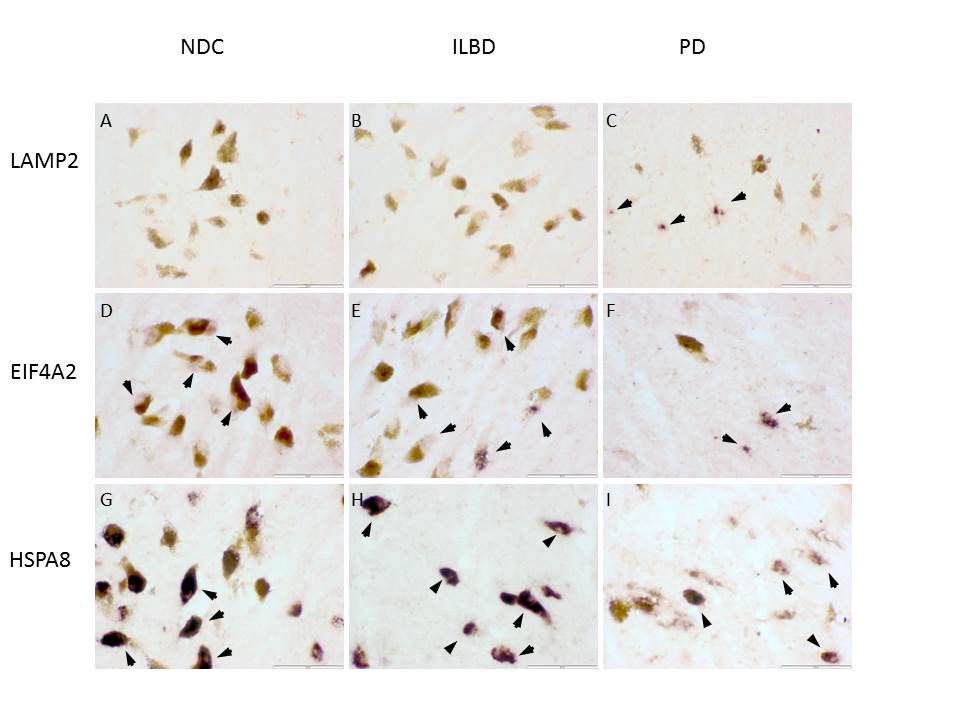

Supplement: S2 Fig — LAMP2 expression (A, B, C) is detected in neuronal cells in PD substantia nigra. EIF4A2 (D, E, F) shows lower expression in the dopaminergic neurons of PD donors compared to iLBD and control cases; HSPA8 (G, H, I) shows strong expression in the dopaminergic neurons in all groups. Arrow heads indicate dopaminergic neurons. (JPG) [file pone.0128651.s008.jpg]
